# Biocontrol Activity of *Trichoderma* Species Isolated from Grapevines in British Columbia against Botryosphaeria Dieback Fungal Pathogens

**DOI:** 10.3390/jof8040409

**Published:** 2022-04-16

**Authors:** Jinxz Pollard-Flamand, Julie Boulé, Miranda Hart, José Ramón Úrbez-Torres

**Affiliations:** 1 Summerland Research and Development Centre, Agriculture and Agri-Food Canada, 4200 Highway 97, Summerland, BC V0H 1Z0, Canada; jinxz.flamand@agr.gc.ca (J.P.-F.); julie.boule@agr.gc.ca (J.B.); 2 Department of Biology, The University of British Columbia Okanagan, 3187 University Way, Kelowna, BC V1V 1V7, Canada; miranda.hart@ubc.ca

**Keywords:** *Vitis vinifera*, biocontrol, *Trichoderma*, grapevine trunk diseases, botryosphaeria dieback, *Diplodia seriata*, *Neofusicoccum parvum*, translation elongation factor one alpha

## Abstract

Botryosphaeria dieback (BD) is a grapevine trunk disease (GTD) causing significant yield losses and limiting the lifespan of vineyards worldwide. Fungi responsible for BD infect grapevines primarily through pruning wounds, and thus pruning wound protection, using either synthetic chemicals or biological control agents (BCAs), is the main available management strategy. However, no products to control GTDs are currently registered in Canada. With a focus on more sustainable grapevine production, there is an increasing demand for alternatives to chemical products to manage GTDs. Accordingly, the objective of this study was to identify *Trichoderma* species from grapevines in British Columbia (BC) and evaluate their potential biocontrol activity against BD fungi *Diplodia seriata* and *Neofusicoccum parvum*. Phylogenetic analyses identified seven species, including *T. asperelloides*, *T. atroviride*, *T. harzianum*, *T. koningii*, *T. tomentosum,* and two novel species, *T. canadense* and *T. viticola*. In vitro dual culture antagonistic assays showed several isolates to inhibit fungal pathogen mycelial growth by up to 75%. In planta detached cane assays under controlled greenhouse conditions identified *T. asperelloides*, *T. atroviride* and *T. canadense* isolates from BC as providing 70% to 100% pruning wound protection against BD fungi for up to 21 days after treatment. In addition, these isolates were shown to provide similar or better control when compared against commercial chemical and biocontrol products. This study demonstrates the potential that locally sourced *Trichoderma* species can have for pruning wound protection against BD fungi, and further supports the evaluation of these isolates under natural field conditions.

## 1. Introduction

Botryosphaeria dieback (BD) is one of several grapevine trunk diseases (GTDs); it occurs wherever grapes are grown and is caused by a wide range of fungal species within the Botryosphaeriaceae family [1]. Some species responsible for BD are known to occur in many different hosts and are thought to be present in an endophytic phase before transitioning to a pathogenic phase under certain abiotic and/or biotic stress conditions [2]. BD fungi are also reported to infect grapevine material during the propagation process and, consequently, can be introduced into vineyards through contaminated ready-to-plant nursery stock [3]. However, Botryosphaeriaceae spp. primarily infect grapes through pruning wounds via air-borne conidia, which are dispersed by rain-splash from pycnidia located on infected wood [4,5,6]. Symptoms of BD are diverse, can be observed in both young and mature grapevines, and mainly include poor vigor and the presence of wedge-shaped cankers in the vascular tissue of spurs, cordons and/or trunks [1]. BD is a destructive disease that causes mortality of plant parts and eventual death of the entire vine, and thus it is responsible for important economic losses [7,8].

To date, no reports of resistance of *Vitis vinifera* L. or *Vitis* spp. to BD fungi are available and tested cultivars have all shown some degree of susceptibility [9,10,11]. Furthermore, apart from ‘renewal surgery’, in which infected parts of the vine are surgically removed [12], no other curative treatment is currently available for vines infected by BD. In the absence of curative treatments, the study of both cultural and preventative treatment measures has long been the focus for scientists seeking to effectively control this disease [13]. Wounds made every year during the pruning season are the main point of infection; therefore, since the mid-1980s, protection of pruning wounds has been the most commonly used and researched control strategy. Protection of pruning wounds to manage BD fungi is not a new approach and studies using this principle have been conducted since the mid-1980s [13]. As a result, a wide range of active chemical ingredients have been screened in both nurseries and commercial vineyards, with many that are currently registered and available to growers in different countries [3,13]. To a lesser extent, attention has been paid to investigating the potential that other natural compounds and/or biological control agents (BCAs) may offer as alternative solutions for pruning wound protection. Among the several generalist and specialist BCAs screened to manage GTDs, including BD fungi, species within *Trichoderma* are by far the most widely studied [13].

*Trichoderma*-based products represent over 60% of registered biopesticides [14], making these fungi the most used BCAs in agricultural crops, including grapevines [15,16]. Species of *Trichoderma* are ubiquitous in soil all over the world and are often the most isolated, with 10^1^–10^3^ culturable propagules per gram of soil in most temperate and tropical forests [17]. Most *Trichoderma* spp. act as opportunistic plant symbionts capable of promoting plant growth while actively antagonizing plant-pathogenic fungi by direct competition for the same environmental niche, which in grapevines can include xylem and parenchyma [17]. In addition, they antagonize via antibiosis, including the secretion of cell-wall-degrading enzymes and antagonistic secondary metabolites, which can cause lysis and hyphal swelling in the prey [18]. These properties have been explored as an alternative to chemicals for the management of GTDs, and several studies have evaluated *Trichoderma* spp. as BCAs against black-foot [19,20], esca [21,22], and Eutypa dieback [23,24]. Moreover, studies are also available on the efficacy that *Trichoderma* spp. have on controlling BD fungi at the nursery level [25,26] or as pruning wound protectants in planta, either under controlled laboratory/greenhouse conditions [27,28,29,30] or under natural environmental conditions in the vineyard [30,31,32]. However, these studies report variable results on the efficacy of *Trichoderma*. For instance, *T. atroviride*-based commercial products significantly reduced pruning wound infection by GTD fungi, including the BD pathogen *D. seriata,* in studies conducted in South Africa [30]. Conversely, *T. atroviride*-based formulations showed very low levels of pruning wound protection against *D. seriata* in field trials conducted in Spain [32]. Most available studies are based on the use of a very narrow number of both *Trichoderma* spp. and isolates, mainly *T. atroviride* and *T. harzianum*, and on many occasions in the form of commercial products previously developed to control different pathogens in crops other than grapevines. This may present a problem when trying to adopt a BCA product from another climate or ecosystem, as it has been reported that the effectiveness of *Trichoderma*-based formulations can vary between in vitro and in situ studies performed in different hosts and under different environmental conditions [14,33]. Accordingly, in recent years, there is increased interest in evaluating the potential that locally isolated endophytic *Trichoderma,* either from grapevines or from other substrates, could have for increasing the efficacy of BCAs against GTD fungi, since they could be better adapted to the local ecosystem. However, few studies have explored this route against GTD fungi [23,27,28,30,31,34].

Viticulture accounts for 35% of all pesticides used worldwide, exceeding four million tons in 2018 [16]. However, as observed in many other crops, chemical fungicides are increasingly restricted and/or regulated, or banned due to concerns related to high toxicity to humans, animals and/or the environment as well as the development of resistance by pathogens. This, along with higher consumer demand for more sustainable agriculture production systems, has caused viticultural practices to experience important changes in recent decades [16]. It is now well-accepted that an integrated pest management approach (IPM), in which the responsible use of synthetic chemicals along with cultural practices and the use of BCAs and/or organic products, is the best option to implement in order to minimize the development and spread of diseases, including GTDs [35].

Grapevines are an emerging crop in Canada. Though it is still relatively a young grape growing country, GTDs have already been identified as an important biotic factor threatening the economic sustainability of the Canadian grape and wine industry [36,37,38]. However, unlike in most grape growing countries around the world, there are currently no registered control products, neither synthetic fungicides nor BCAs, for the management of GTDs in Canada. This highlights the urgent need to generate data and develop strategies to help manage these destructive diseases to support an economically important and growing industry [39]. In addition, with the growing trend towards developing a more sustainable Canadian grape and wine industry, there is an important need to provide biological control alternatives against these diseases. Accordingly, the main objectives of this study were to (i) identify and characterize *Trichoderma* spp. locally isolated from vineyards in BC by means of morphological and molecular studies, and (ii) determine their potential as BCAs against the botryosphaeriaceous taxa *D. seriata* and *Neofusicoccum parvum*, two of the most widespread GTD fungi infecting grapevines in British Columbia (BC).

## 2. Materials and Methods

### 2.1. Trichoderma Isolates and Botryosphaeria Dieback Fungal Pathogens

A total of 29 *Trichoderma* isolates, obtained from vineyard field surveys conducted in the Okanagan Valley, BC between 2011 and 2018, were used for this study (Table 1). *Trichoderma* isolates were primarily isolated from roots and the basal end of either rootstock or self-rooted plants from both young and mature vineyards, as previously described [37]. Pure cultures were obtained by hyphal tip on potato dextrose agar (PDA, DIFCO™, Detroit, MI, USA) and *Trichoderma* isolates were stored as colonized PDA agar plugs in glass vials (15–20 plugs/vial) containing double-autoclaved distilled water at 4 °C, at the Summerland Research and Development Centre (SuRDC), Summerland, BC fungal collection, until testing. When needed, isolates were retrieved from the collection and revived by plating three to five colonized agar plugs into fresh 90 mm-diameter PDA Petri plates. Plates were wrapped in Parafilm and incubated in the dark at 23 °C until colonies were observed. Single colonies were obtained by transferring the edge of an active growing colony onto new PDA plates. Two BD pathogens, *D. seriata* (SuRDC-1089) and *N. parvum* (SuRDC-1064), were selected for this study as they represent two of the most prevalent canker-causing fungi isolated from grapevine showing BD symptoms in the Okanagan Valley. Both isolates were proven to be pathogenic and highly virulent [38]. Isolates were stored and revived from the SuRDC fungal collection, as previously described.

### 2.2. Identification of Trichoderma Isolates

#### 2.2.1. DNA Extraction, PCR Amplification and Sequencing

Total DNA was extracted from pure cultures of all 29 isolates by collecting 0.1 g aerial mycelia from colonies actively growing on PDA using the Power Soil DNA Isolation Kit (MO BIO Laboratories Inc., Carlsbad, CA, USA) following the manufacturer’s instructions. Polymerase Chain Reaction (PCR) was used for all isolates to amplify the internally transcribed spacer region (ITS), including the 5.8S ribosomal gene as well as part of the translation elongation factor 1 alpha gene (*TEF1*), using ITS1/ITS4 and TEF71F/TEF997R primers, respectively [40,41]. PCR reactions consisted of 13.85 µL NF H_2_O, 2 µL 10× Buffer, 2 µL dNTPs (2.0 mm), 0.4 µL Blotto 10% *w*/*v*, 0.25 µL forward primer (20 µm), 0.25 µL reverse primer (20 µm), 0.25 µL DreamTaq (ThermoFisher Scientific, Burlington, ON, Canada), and 1 µL sample DNA. PCR amplifications were performed using a GeneAmp 2700 thermal cycler (Applied Biosystems, Foster City, CA, USA) under the conditions previously described [40,41]. All PCR products were purified using a QIAquick PCR purification Kit (QIAGEN Inc., Valencia, CA, USA), and both strands of the ITS and *TEF1* were sequenced using a 8-capillary AB 3500 Genetic Analyzer Sanger Sequencer (Foster City, CA, USA) at the SuRDC.

#### 2.2.2. Trichoderma Species Identification and Phylogenetic Analyses

Forward and reverse nucleotide sequences from each *Trichoderma* isolate were assembled, proofread, and edited using Lasergene SeqMan Pro version 17.2.0 (DNASTAR Inc., Madison, WI, USA). Consensus sequences were then compared with those available in the GenBank database using the Basic Local Alignment Search Tool (BLASTn) function, and homologous sequences with high similarity were recorded. *TEF1 Trichoderma* sequences from BC were separately aligned with published GenBank sequences, including ex-type specimens when available, using BioEdit Sequence Alignment Editor Version 7.1.3.0 computer software [42]. Alignments were inspected visually and edited manually in BioEdit. Two separate phylogenetic studies including *TEF1* sequences from the *viride*/*viridescens* and *viride*/*koningii* species groups [41] were conducted using MEGA-X [43]. The best-fit substitution model was determined in MEGA-X in order to be used in the phylogenetic evolutionary analyses. All datasets were analyzed using two different optimality search criteria, the maximum likelihood (ML) method and the Tamura–Neil model, with 1000 replicates to assess robustness and maximum parsimony (MP) with the bootstrap test (1000 random additional sequence replicates) and the Tree-Bisection-Regrafting (TBR) algorithm. Phylogenetic trees with the greatest log likelihood were selected. *Trichoderma* sequences from BC were deposited into GenBank, and isolates are maintained at the SuRDC fungal collection and at the Department of Agriculture Ottawa Mycology Collection (DAOM), Ottawa (ON).

#### 2.2.3. Morphological Characterization of Novel Trichoderma Species

Isolates SuRDC-1422 and SuRDC-1430, representing novel identified *Trichoderma* spp. in this study, were grown on PDA Petri plates at room temperature (~22 °C) for 14 days in order to describe colony morphological characteristics, including colony growth and type and mycelium color using the color charts of Rayner (1970) [44]. Pictures of colonies were taken after three, seven, and fourteen days using a Nikon D700 60 mm. Morphological characteristics of microscopic structures such as mycelium, conidia, conidiophores, and phialides were noted after seven days of incubation on PDA at room temperature under laboratory lighting conditions. Conidial suspensions were obtained by adding 1 mL sterile distilled water amended with Tween 20 (~1 drop per 300 mL) and gently scraping the aerial mycelia to suspend conidia. The suspension was then filtered through a sterile cotton ball before mounting on microscope slides. Reproductive structures were harvested using sterile forceps and mounted on microscope glass-slides. Structures were observed and photographed using Zeiss Axio Imager.M2 with a Zeiss AxioCam MRm at 40× magnification, using bright field and differential interference contrast. Photographs were taken using the program Zeiss ZenPro. Fifty conidia were measured per isolate. Length and width of conidia, phialides and conidiophores were measured using the ImageJ software. Phialide widths were measured at the widest part of the flask base. The maximum and minimum Feret diameters were measured for each conidium and the means were calculated for each isolate.

#### 2.2.4. Trichoderma Optimum Mycelial Growth Study

At most, two isolates of each *Trichoderma* sp. identified from the molecular studies were selected to determine their optimum temperature for radial mycelial growth. Seven-millimeter plugs were taken from the edges of six-day-old actively growing *Trichoderma* colonies on PDA. One plug per isolate was placed at the edge of a 90 mm Petri dish with a black line drawn through the center of the bottom of the plate containing 20 mL of PDA pipetted with a 50 mL disposable pipette and a powerpette plus pipette controller (Jencons Scientific, Leighton Buzzard, UK). Petri plates were Parafilmed and placed in their respective incubators at eight temperatures, at 5 °C intervals from 5 to 40 °C, in the dark. Radial growth of each isolate was measured after four days of incubation. Each isolate/temperature combination was performed in triplicate and the entire experiment was repeated. A paired samples *t*-test was used to test for statistical differences between the first and the repeated experiment to determine if data from separate experiments could be pooled for analysis. The mean radial mycelial growth and standard error for each temperature/species combination were calculated. Statistical analyses were performed using R [45].

### 2.3. Dual Culture Antagonism Assay

Twenty-six of the twenty-nine isolates were selected to determine the antagonistic capability of *Trichoderma* spp. identified in BC against the botryosphaeriaceous fungi *D. seriata* (SURDC-1089) and *N. parvum* (SURDC-1064) by conducting a dual culture antagonism assay (DCAA), adapted from a previously described method [28]. The only *T. tomentosum* isolate identified in this study (SuRDC-1431), as well as *T. canadense* isolates SuRDC-1435 and SuRDC-1451, did not survive in the culture collection and therefore were not included in this assay. Briefly, seven-millimeter diameter mycelial plugs of each *Trichoderma* and pathogen were obtained from the edge of five-day-old active growing colonies on PDA and placed opposite to each other at the edges of 90 mm diameter Petri dishes containing 20 mL PDA. *Diplodia seriata* and *N. parvum* isolates were individually grown separately without the presence of *Trichoderma* as positive controls. Colonies were incubated in the dark at 23 °C for a total of seven days. Colonies were left to grow for two days before pathogen colony radii were measured for the next five days at the same time each day. Measurement was performed from the edge of the plate to the edge of the colony or to the most distinguishable border between the *Trichoderma* spp. and the pathogen. Colonies were removed from incubators for no more than one hour each day for measurement of colony radii. All pairings were performed in triplicate and the experiment was repeated. The percentage of mycelium inhibition was calculated using the formula:

$$\mathrm{Percent}\;\mathrm{Inhibition}\;\left(\%\right)=\frac{b-a}{b}\times 100$$

where *a* is the radius of pathogen mycelial growth co-inoculated with *Trichoderma* and *b* is the radius of the pathogen mycelial growth alone in the control plate measured on the seventh day post-inoculation. The experimental data from the DCAA were first subjected to an analysis of variance (ANOVA) followed by Tukey’s test (*p* = 0.05) to determine if there was a statistical difference between the first and second experimental repeat and therefore if data could be pooled. Data were then subjected to ANOVA followed by Tukey’s test (*p* = 0.05) to test for differences between the isolates. The standard error was calculated for all mean values. All statistical analyses were performed using R.

### 2.4. Detached Cane Assays

#### 2.4.1. Trichoderma and Pathogen Inoculum Preparation

*Trichoderma* inoculum from each isolate used in this experiment was obtained from four-day-old colonies grown on PDA at 23 °C with a 12:12 h per day photoperiod under UV light (Phillips UVB TL 20W/12RS bulb, Meditec-Plus Inc., Rosemere, QC, Canada). Plates were flooded with sterile distilled water (SDW) containing 0.05% Tween 20 and surfaces were scraped with a sterile metal spatula. Water was then filtered through one layer of autoclaved 25 µm pore diameter Miracloth (Sigma-Aldrich Canada Co., Oakville, ON, Canada) to remove mycelium fragments. Spore suspensions of each *Trichoderma* isolate were adjusted to 1 × 10^6^ spores/mL with a haemocytometer. Similarly, *D. seriata* and *N. parvum* conidia were obtained from pycnidia formed on colonies growing on PDA in the dark at 25 °C under UV light (12:12 h) placed at 80 cm distance from the plates. Pycnidia formed after 4–5 weeks and conidia were harvested by adding 1–2 mL of SDW amended with a drop of Tween 20, and then gently scraping the upper layer of the PDA to detach the pycnidia from the agar using a sterile metal spatula. Suspensions containing pycnidia were transferred onto a sterile mortar and pestle and ground to release spores from pycnidia. The suspensions were then filtered through one layer of Miracloth into a 20 mL glass Pyrex test tube. Tubes were vortexed and spores concentrations adjusted to 1 × 10^5^ spores/mL for both *D. seriata* and *N. parvum* using a haemocytometer.

#### 2.4.2. Trichoderma Species Detached Cane Assay

The biocontrol activity of the best-performing isolates from the DCAA was evaluated in planta under controlled greenhouse conditions against *D. seriata* (SURDC-1089) and *N. parvum* (SURDC-1064) via detached cane assay (DCA) modified from Ayres et al. (2011) [46]. Dormant Chardonnay canes were collected from an experimental vineyard block located at the SuRDC. Canes were cut into two-node canes (~20 cm length) and placed vertically through holes created in Styrofoam trays floating on water tables filled with tap water in a greenhouse. Temperature and relative humidity (RH) were monitored in the greenhouse over the duration of the experiment using a Hygrochron I-button (IButtonLink, LLC, Whitewater, WI, USA). The water level was maintained throughout the experiment by adding tap water; however, total water was not exchanged. Care was taken to ensure that the bottoms of the canes were submerged in water for the duration of the experiment. The best-performing isolates from the DCAA were selected for this experiment. Canes were then pruned ~4 cm above the upper bud to simulate a fresh pruning wound. A total of 270 canes were prepared per treatment. Within 3 h after pruning, 180 canes (pruning wounds) per treatment were inoculated with 50 µL of 1 × 10^6^ spores/mL (50,000 spores/wound) of the selected *Trichoderma* isolates using a micropipette. The rest of the 90 canes from each treatment were left untreated as positive and negative controls. In each treatment group, 30 canes (3 replicates of 10 repetitions each) were challenged 24 h, 7 d and 21 d after treatment with *Trichoderma* with 50 µL of 1 × 10^5^ conidia/mL (5000 spores/wound) of either *D. seriata* or *N. parvum* to determine *Trichoderma* protection activity over time on the pruning wound. Positive controls included 30 non-treated but inoculated canes with 5000 spores/wound of *D. seriata* or *N. parvum*. Control treatments were inoculated once at either 24 h, 7 d, or 21 d after pruning. In addition, 30 canes per treatment were left non-treated and non-inoculated to serve as negative controls to determine if natural infections happened in the canes collected from the experimental vineyard. Canes from the different treatments were randomized across the Styrofoam trays. Cuttings were maintained in the greenhouse and collected five weeks after each of the pathogen inoculation times.

#### 2.4.3. Commercial Products Detached Cane Assays

Commercial control products (chemical and biological) were also included in a separate DCA to test their effectiveness in protecting pruning wounds against artificial infection by *D. seriata* and *N. parvum,* and to compare against *Trichoderma* spp. identified in this study (Table 2). The methodology followed for this DCA was the same as described for the *Trichoderma* DCA experiment and dormant Chardonnay canes were also used. The same number of canes per treatment was used, as described above. Pruning wounds were treated within 3 h after pruning with the different commercial products following the label recommendations (Table 2). Tebuconazole and *Trichodema*-based products were applied using a paintbrush, making sure that the entire pruning wound was covered. Tetraconazole was prepared in 1 L handheld spray bottles and applied over the pruning wounds until runoff. Thirty canes each (3 replicates of 10 repetitions each) were then challenged 24 h, 7 d and 21 d after treatment with 5000 spores/wound of either *D. seriata* or *N. parvum* to determine the commercial products’ protection activity over time on pruning wounds. The same number of both positive and negative controls was included, as previously described. Canes from the different treatments were randomized across the Styrofoam trays, maintained in the greenhouse and collected five weeks after each of the pathogen inoculation times.

#### 2.4.4. Pathogen Recovery

Roots and leaves were removed from collected canes before the canes were prepared for re-isolation of the inoculated pathogens. Fungal re-isolations started with first shaving the bark around the pruning wound, then flame sterilizing the surface of the cane with 95% ethanol. An approximately 1 mm disc section was removed. An approximately 2 mm disc section was removed and ten pieces of tissue (~0.5 cm^2^ each) were cut from the section and plated on PDA amended with 1 mg/mL tetracycline (PDA-tet; Sigma-Aldrich; St. Louis, MO, USA). Plates were incubated for up to 10 days at 23 °C in the dark. If a plate yielded either *D. seriata* or *N. parvum*, the corresponding cane was rated as colonized by the pathogen. Treatment efficacy was based on the mean percent recovery (MPR) of *D. seriata* and *N. parvum* from treated canes, and data are presented as mean percent disease control (MPDC) according to the formula:

$$\mathrm{MPDC}=100\times \left(1-\frac{\mathrm{MPR}\;\mathrm{treatment}}{\mathrm{MPR}\;\mathrm{control}}\right)$$

The binary (infected or non-infected) data produced from the DCA were subjected to ANOVA followed by Tukey’s HSD post-hoc comparison test to determine if there were significant statistical differences between the experimental means (*p* = 0.05). All statistical analyses were performed in R.

#### 2.4.5. Identity Confirmation of Re-Isolated *Trichoderma* spp. and Fungal Pathogens

In order to confirm that the *Trichoderma* spp. re-isolated from the treated canes were the same species inoculated in the initial treatment, pure cultures of the re-isolated *Trichoderma* spp. were obtained by hyphal tipping (one representative isolate per treatment). Total genomic DNA was extracted as previously described. PCR amplification using ITS1/ITS4 primers was followed by Sanger sequencing, and consensus sequences were aligned with the original ITS sequence obtained from of each *Trichoderma* isolate used. Identity confirmation for re-isolated BD pathogens *D. seriata* and *N. parvum* was conducted by morphological comparisons against pure cultures of the original isolate used.

## 3. Results

### 3.1. Identification of Trichoderma Isolates from Grapevines from British Columbia

PCR amplifications of the ITS and *TEF1* regions generated, respectively, fragment products of 555 to 624 bp and 705 to 889 bp. A primary identification of both ITS rDNA and *TEF1* sequences was performed using BLASTn in the GenBank database. Results assigned the 29 *Trichoderma* isolates obtained in this study to seven different taxa (Table 1). ITS and *TEF1* sequences from 22 isolates showed 99% to 100% homology with five *Trichoderma* spp. from GenBank, including ex-type specimens, which were *T. asperelloides* (four isolates), *T. atroviride* (seven isolates), *T. harzianum* (eight isolates), *T. koningii* (two isolates), and *T. tomentosum* (one isolate). The ITS rDNA sequences of four (SuRDC-1422, SuRDC-1435, SuRDC-1450, and SuRDC-1451) and three isolates (SuRDC-1427, SuRDC-1429, and SuRDC-1430) showed 99% to 100% homology with *T. koningiopsis* and *T. viride* isolates, respectively, from GenBank. However, the *TEF1* sequence of isolates SuRDC-1422, SuRDC-1435, SuRDC-1450 and SuRDC-1451 showed only 95% to 97% homology with *T. koningiopsis* isolates from GenBank. Similarly, when BLASTn was conducted for *TEF1* sequences of isolates SuRDC-1427, SuRDC-1429, and SuRDC-1430, only five *T. viride* isolates from GenBank matched our isolates with 97% homology. Therefore, these isolates were thought to represent two possible novel phylogenetic species.

**Figure 1:**
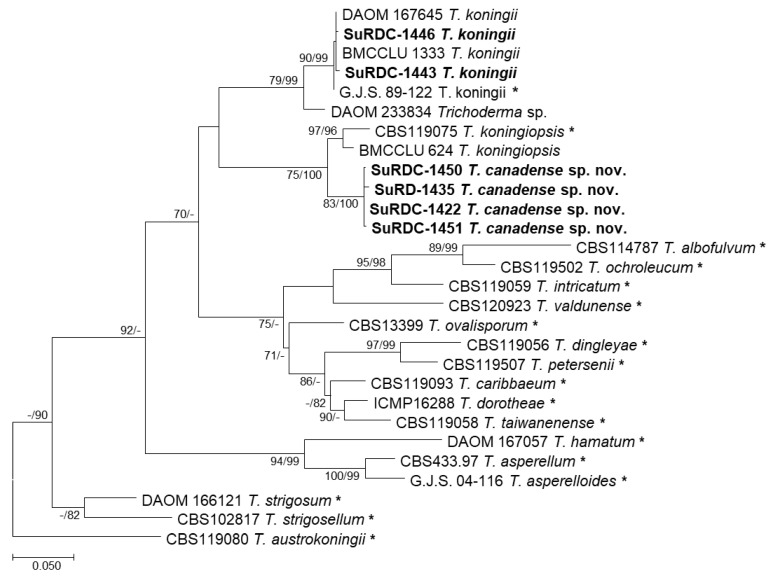
Phylogram of the highest log likelihood tree from an analysis of the *TEF1 viride*/*koningii* dataset. Numbers in front of and after the slash represent ML and MP bootstrap values from 1000 replicates, respectively. Values represented by a dash (-) were less than 70% for the bootstrap analyses. Type specimens are indicated with an asterisk and *Trichoderma* isolates from vineyards in BC are indicated in bold. Bar indicates the number of substitutions per site.

Two separate *TEF1* phylogenetic analyses were conducted to further support the discovery of the abovementioned novel phylogenetic species. The first analysis involved 28 nucleotide sequences, including isolates SuRDC1422, SuRDC-1435, SuRDC-1450 and SuRDC-1451 from BC, and there were a total of 942 positions in the final dataset. Isolates from BC were compared against closely related type specimens representing phylogenetic species in the *viride*/*koningii* clade as previously described by Braithwaite et al. (2017) [41]. *Trichoderma austrokoningii* isolate CBS119080 was selected as the outgroup. ML analyses and the Hasegawa–Kashino–Yano model yielded the highest likelihood tree (−5709.96), which is shown in Figure 1. A discrete Gamma distribution was used as the best-fit model for evolutionary rate differences among sites (five categories (+G, parameter = 3.5291)), as previously described in MEGA-X. Maximum parsimony analyses of these taxa yielded two most parsimonious trees (length = 966) with similar topologies to the highest log likelihood tree. Consistency index, retention index, and composite index from the MP analyses were 0.629534, 0.747795 and 0.526398, respectively. Bootstrap branch support (ML/MP) determined by 1000 replicates is shown in Figure 1. Isolates SuRDC1422, SuRDC-1435, SuRDC-1450, and SuRDC-1451 formed a strongly supported single lineage (89/100) closely related to *T. koningiopsis,* confirming these isolates to be a novel species named *T. canadense* (Figure 1). Taxonomic descriptions of this species follow.

**Figure 2:**
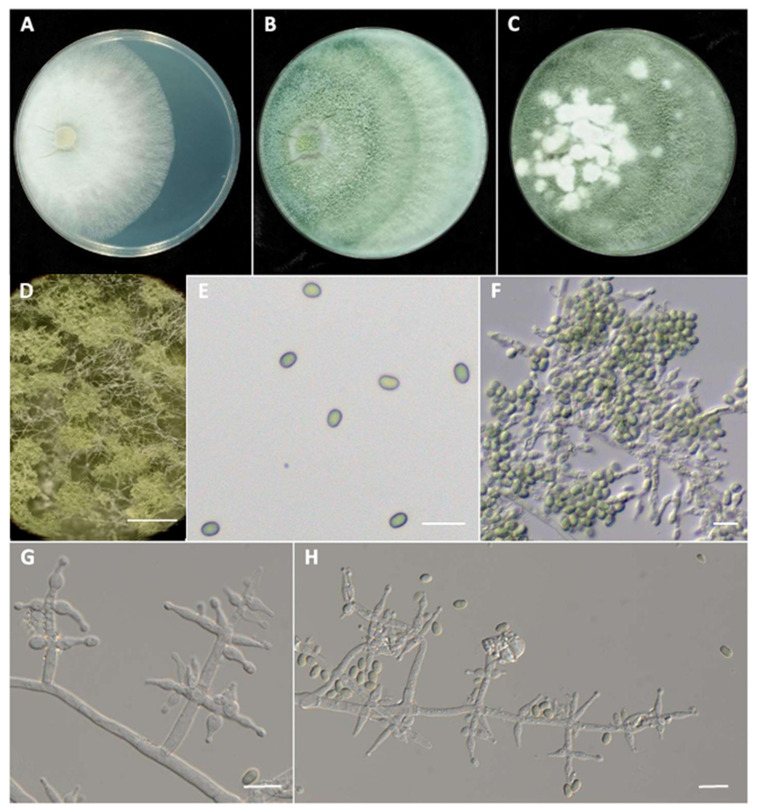
*Trichoderma canadense* SuRDC-1422. Cultures on PDA at ~22 °C after 3 d (**A**), 7 d (**B**), and 14 d (**C**). Conidiation pustules on PDA after 14 d (**D**). Single conidia (**E**) and mass conidia (**F**). Conidiophores and phialides (**G**,**H**). Scale bars represent 1 mm (**D**) and 10 µm (**E**–**H**).

***Trichoderma canadense*** J. Pollard-Flamand & J.R. Úrbez-Torres sp. nov. (Figure 2)

Mycobank# 843655 

*Etymology.* Named after Canada, where this species was collected.

Minimum temperature for growth 5 °C, optimum 25 °C, maximum 35 °C. On PDA after 96 h, colony radius 6.3 mm at 5 °C, 83 mm at 25 °C and 6.7 mm at 35 °C. Colonies on PDA initially white, regularly circular, distinctly zonate with dense mycelium in the center and slightly loose at the margin. Aerial hyphae loose. No diffusing pigment (Figure 2). Colony turned green in the center after 5 d, expanding gradually with time towards the margin (Figure 2). Conidial production noted after 7 d at (~22 °C), starting around the inoculum.

Mycelium consisting of branched, septate hyaline hyphae that occur singly 3.5–7.5 µm wide. Conidiophores on PDA mostly aggregated, but after approximately 7 d formed compact pustules up to 1.5 mm diameter (Figure 2). Pustules clearly observed around the inoculum after 14 days, first white and turning green with age, and hyphae protruding beyond the surface 0.5–0.8 mm in diameter. Conidiophores developing predominantly as branches from the aerial mycelium, pyramidal, main axis coarse, and straight (up to 137.6 µm long). Branches predominantly straight, thinner than main axis, paired at irregular intervals. Terminal cells circular to ellipsoidal. Phialides hyaline, obpyriform to langeniform, occasionally curved from terminal conidiophore branches or arising singly along the sides of the conidiophore (4.5–)9.8–10.6(–16.6) × (2.9–)4.5–4.7(–6.5) µm, length/width ratio = 2.6 (*n* = 50). Conidia cylindrical with rounded ends, often ellipsoidal or subglobose (3.9–)4.8–5.2(–5.9) × (3.2–)3.7(–4.6) µm, length/width ratio = 1.3, average area 13.8 µm (*n* = 50), thin-walled, singly pale yellow, light green in mass (Figure 2). Chlamydospores unobserved. Teleomorph undetermined.

*Type:* Canada, British Columbia, Okanagan Valley, isolated from vascular tissue (xylem) at the basal end of ‘Riparia gloire’ rootsock (*Vitis riparia*) grafted onto ‘Merlot’ (*Vitis vinifera*), J.R. Úrbez-Torres: SuRDC-1422.

*Additional Material Examined:* Canada, British Columbia, Okanagan Valley, J.R. Úrbez-Torres: isolated from vascular tissue (xylem) at the basal end of ‘Riparia gloire’ rootsock grafted onto Pinot Noir, SuRDC-1435, roots SuRDC-1450, and graft-union SuRDC-1451. 

*Sequences:* SuRDC-1422 (MZ161796, ITS, MZ189377, *TEF*), SuRDC-1435 (MZ161797, ITS, MZ189378, *TEF*), SuRDC-1450 (MZ161798, ITS, MZ189379, *TEF*), SuRDC-1451 (MZ161799, ITS, MZ189380, *TEF*).

*Notes: Trichoderma canadense* was isolated four times from the xylem tissue of four different young grapevines in the current study. *Trichoderma canadense* can be readily distinguished from known *Trichoderma* spp. by phylogenetic analyses of the *TEF1* sequences. Phylogenetic analyses revealed *T. canadense* to be closely related to *T. koningiopsis*.

**Figure 3:**
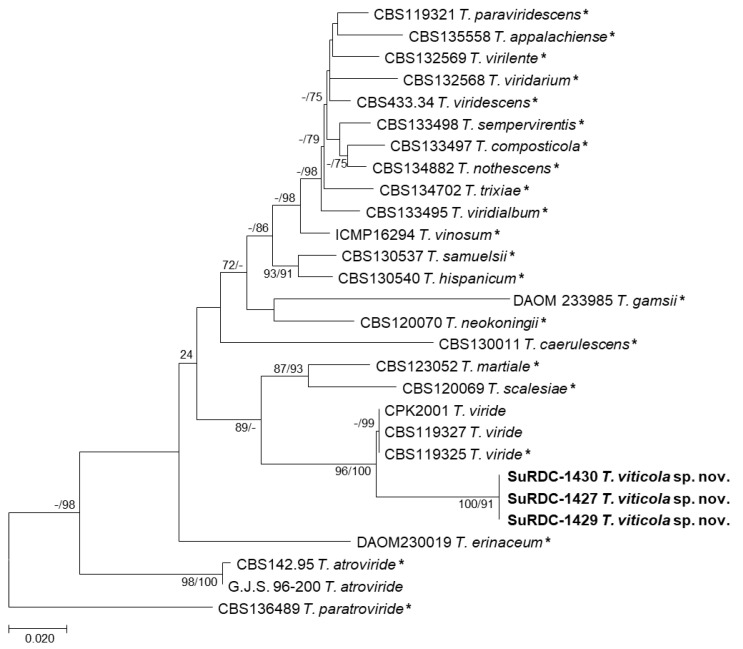
Phylogram of the highest log likelihood tree from analyses of the *TEF1 viride*/*viridescens* dataset. Numbers in front of and after the slash represent ML and MP bootstrap values from 1000 replicates, respectively. Values represented by a dash (-) were less than 70% for the bootstrap analyses. Type specimens are indicated with an asterisk and *Trichoderma* isolates from vineyards in BC are indicated in bold. Bar indicates the number of substitutions per site.

The second analyses included 28 nucleotide sequences, including isolates SuRDC-1427, SuRDC-1429, and SuRDC-1430 from BC, and there were a total of 1642 positions in the final dataset. Isolates from BC were compared against closely related type specimens representing phylogenetic species in the *viride*/*viridescens* clade as previously described by Braithwaite et al. (2017) [41]. *Trichoderma paratroviride* isolate CBS136489 was selected as the outgroup. ML analyses and the Hasegawa–Kashino–Yano model yielded the highest likelihood tree (−5903.77), which is shown in Figure 3. A discrete Gamma distribution was used as the best-fit model for evolutionary rate differences among sites (five categories (+G, parameter = 0.2670)) as previously described in MEGA-X. Maximum parsimony analyses of these taxa yielded four most parsimonious trees (length = 717) with similar topologies to the highest log likelihood tree. Consistency index, retention index, and composite index from the MP analyses were 0.603104, 0.761651, and 0.571504, respectively. Bootstrap branch support (ML/MP) determined by 1000 replicates is shown in Figure 3. Isolates SuRDC1427, SuRDC-1429, and SuRDC-1430 formed a strongly supported single lineage (100/91) closely related to *T. viride* isolates, confirming these isolates to be a novel species named *T. viticola* (Figure 3). Taxonomic descriptions of this species follow hereinafter.

**Figure 4:**
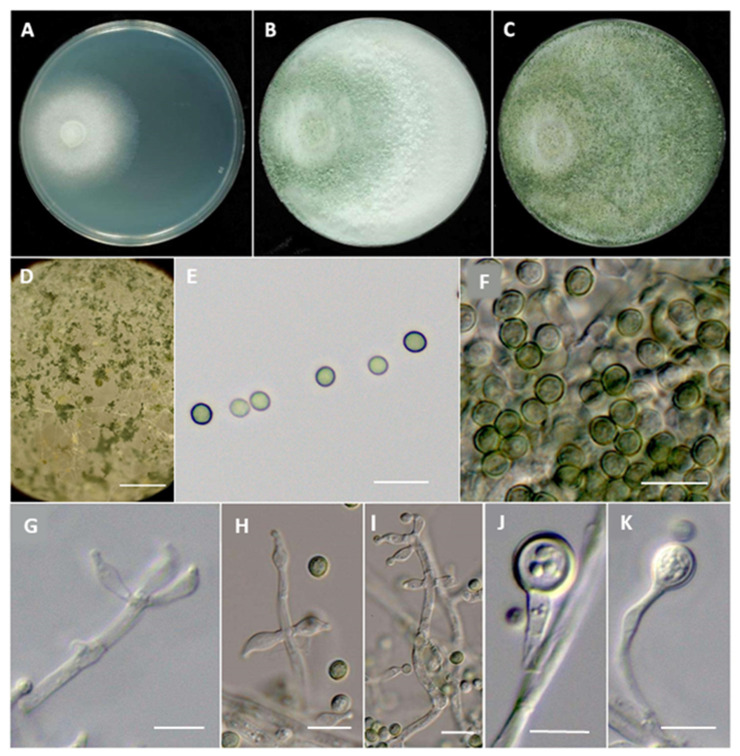
*Trichoderma viticola* SuRDC-1430. Cultures on PDA at ~22 °C after 3 d (**A**), 7 d (**B**), and 14 d (**C**). Conidiation pustules on PDA after 7 d (**D**), and 14 d (**E**). Single conidia (**F**) and mass conidia (**G**). Conidiophores and phialides (**H**–**J**). Chlamydospores (**K**). Scale bars represent 1 mm (**D**) and 10 µm (**E**–**K**).

***Trichoderma viticola*** J. Pollard-Flamand & J.R. Úrbez-Torres sp. nov. (Figure 4).

MycoBank# 843656

*Etymology.* Named after the host it was isolated from, *Vitis vinifera*.

Minimum temperature for growth 5 °C, optimum 20 °C, maximum 35 °C. On PDA after 96h, colony radius 9.3 mm at 5 °C, 62.3 mm at 20 °C and 6.3 mm at 35 °C. Colonies on PDA initially white, regularly circular, zonate with dense cottony mycelium in the center becoming loose towards the margin. Aerial hyphae loose. No diffusing pigment (Figure 4). Colony white with dense mycelium until fully covered Petri plate and gradually turning light green (center) to dark green (margin) after 3 d (Figure 4). Co nidial production noted after 3 d at approximately 22 °C under lab lighting conditions, starting around the inoculum.

Mycelium consisting of branches, septate hyaline hyphae that occur singly 2–4 µm wide. Conidiophores on PDA mostly aggregated, forming loose pustules first becoming compact up to 0.1–0.3 mm diameter (Figure 4). Small and loose pustules formed around the inoculum, becoming pale cream and compact after 14 d with hyphae protruding beyond the surface (Figure 4). Conidiophores developing predominantly as branches from the aerial mycelium, main axis coarse and straight (up to 72.2 µm long). Conidiophores primarily unbranched and often terminating in a whorl of two or three phialides (Figure 4). Terminal cells circular to subglobose. Phialides ampulliform to langeniform, occasionally curved and often constricted below the tip to form a narrow neck (Figure 4). Phialides arising singly, paired and at irregular intervals along the axis of the conidiophore (5.4–)7.7–8.1(–11.6) × (2.3–)3.1–3.3(–5.5) µm, length/width ratio = 2.4 (*n* = 50). Conidia subglobose (3.9–)4.4(–5.1) × (3.3–)4.0(–4.7) µm, length/with ratio = 1.1, average area 13.2 µm (*n* = 50), thin-walled, granular, hyaline when young and becoming light green with age, dark green in mass (Figure 4). Chlamydospores present, thick-walled, spherical, granular, single, arising from hyphae. Teleomorph undetermined.

*Type*: Canada, British Columbia, Okanagan Valley, isolated from vascular tissue (xylem) at the basal end of ‘Riparia gloire’ rootsock (*Vitis riparia*) grafted onto Pinot Meunier (*Vitis vinifera*), J.R. Úrbez-Torres: SuRDC-1430.

*Additional Material Examined:* Canada, British Columbia, Okanagan Valley, J.R. Úrbez-Torres: isolated from vascular tissue (xylem) at the basal end of ‘3309 Couderc’ rootsock (*Vitis riparia* x *V. rupestris*) grafted onto New York Muscat (Muscat Hamburg x Ontario), SuRDC-1427, basal end of ‘Riparia gloire’ rootsock grafted onto Pinot Meunier SuRDC-1429.

*Sequences*: SuRDC-1427 (MZ161791, ITS, MZ189372, *TEF*), SuRDC-1429 (MZ161792, ITS, MZ189373, *TEF*), SuRDC-1430 (MZ161793, ITS, MZ189374, *TEF*).

*Notes*: *Trichoderma viticola* was isolated three times from the xylem tissue of three different young grapevines in the current study. *Trichoderma viticola* can be readily distinguished from known *Trichoderma* spp. by phylogenetic analyses of the *TEF1* sequences. Phylogenetic analyses revealed *T. viticola* to be closely related to *T. viride*.

#### Trichoderma Optimum Mycelial Growth Temperature Study

A Student’s *t*-test showed no significant difference in the means between the first and second repeated experiments (*p* < 0.05), so data from the two experiments were pooled. All *Trichoderma* isolates from BC identified in this study grew in the range of 10–30 °C after four days of incubation in PDA. *Trichoderma koningii*, *T. tomentosum*, and *T. viticola* isolates, along with one isolate of *T. atroviride* (SuRDC-1440) and *T. harzianum* (SuRDC-1439), showed slight growth at 5 °C. All species showed some growth at 35 °C with the exception of *T. harzianum*, *T. tomentosum*, and *T. viticola,* and none of the isolates grew at 40 °C. Optimum temperature for mycelial growth varied among species. The temperatures at which each *Trichoderma* spp. reached the maximum radial growth were 20 °C for *T. atroviride* and *T. viticola*, 25 °C for *T. asperelloides*, *T. canadense*, *T. koningii* and *T. tomentosum*, and 25–30 °C for *T. harzianum*.

### 3.2. Dual Culture Antagonism Assay

**Figure 5:**
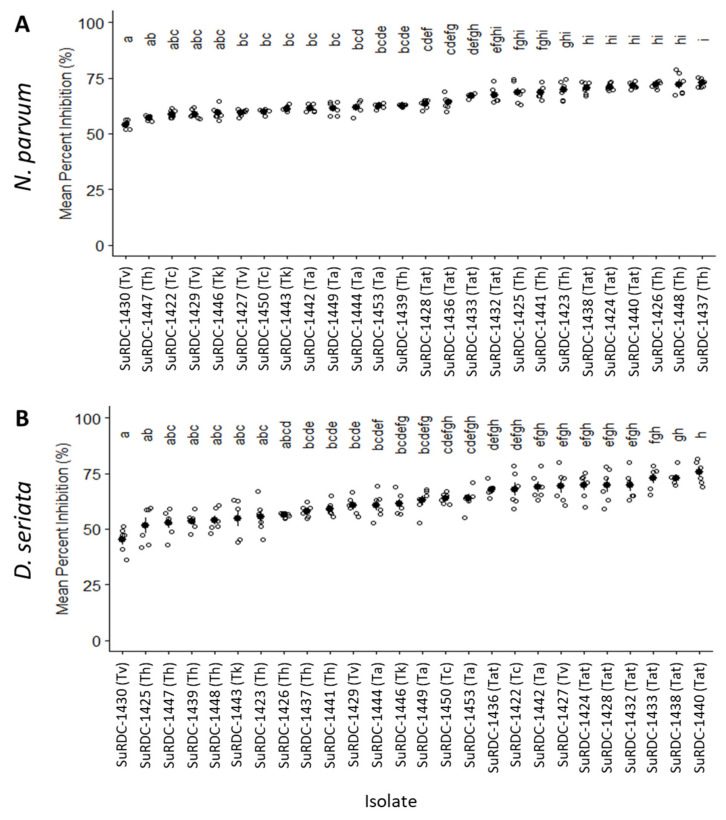
Mean percent radial growth inhibition (MPRGI) of *Trichoderma* isolates from BC against BD pathogens *Neofusicoccum parvum* SuRDC-1089 (**A**) and *Diplodia seriata* SuRDC-1064 (**B**) measured after 5 d. White dots represent data from experiment 1 and repeated experiment 2. Solid black dots represent the MPRGI for each isolate calculated from six replicates. Bars represent standard errors of the means. Columns accompanied by the same letter were determined not to be statistically different by the Tukey–Kramer Honest Significant Difference post-hoc test (*p* = 0.05). Ta, *T. asperelloides*; Tat, *T. atroviride*; Tc, *T. canadensis*; Th, *T. harzianum*; Tk, *T. koningii*; Tv, *T. viticola*.

A Student’s *t*-test showed no significant difference in the means between the first and second repeated experiments (*p* < 0.05), so data from the two experiments were pooled. The level of antagonism of *Trichoderma* isolates from this study against BD pathogens *D. seriata* and *N. parvum* is shown in Figure 5. The mean percent radial mycelial growth inhibition (MPRGI) of *D. seriata* and *N. parvum* by *Trichoderma* isolates from BC selected for this experiment ranged from 45.2% to 75.5%. The highest MPRGI values for *D. seriata* (72.6%) and *N. parvum* (75.5%) were recorded with *T. harzianum* (SuRDC-1437) and *T. atroviride* (SuRDC-1440) isolates, respectively (Figure 5). With the exception of *T. viticola* isolate SuRDC-1430, which showed 45.2% MPRGI when paired against *N. parvum*, the rest of the isolates showed MPRGI higher than 50% against both *D. seriata* and *N. parvum*. *T. atroviride* isolates showed the highest overall MPRGI against *D. seriata*. Both *T. atroviride* and *T. harzianum* isolates showed the highest overall MPRGI against *N. parvum* (Figure 5).

### 3.3. Detached Cane Assays

#### 3.3.1. Trichoderma Species Detached Cane Assays

Results from the DCA greenhouse trial assessing selected *Trichoderma* isolates from BC are shown in Tables 3 and 4. The average T and RH values for this DCA were determined to be 17.7 °C and 38% for the duration of the experiment in the greenhouse. MPR of *D. seriata* and *N. parvum* from positive controls inoculated with 5000 spores/wound were higher than 80% when wounds were inoculated 1 d, 7 d, and 21 d post-treatment. *Diplodia seriata* and *N. parvum* were not isolated from the non-treated and non-inoculated (natural infection) negative controls. All *Trichoderma* isolates from BC, with the exception of *T. viticola* (SuRDC-1427) for *D. seriata*, provided an increase in MPDC over time for both pathogens, with the highest values observed at 21 days post-treatment (Tables 3 and 4). *Trichoderma atroviride* (SuRDC-1440), *T. asperelloides* (SuRDC-1442), and *T. harzianum* (SuRDC-1437) provided the highest MPDC (60–100%) against *D. seriata* from 1 to 21 days post-treatment. The rest of the isolates provided a MPDC range between 10% and 87% when pruning wounds were challenged with *D. seriata* 1 to 21 days post-treatment (Table 3). *Trichoderma atroviride* (SuRDC-1440) provided the highest MPDC (100%) against *N. parvum* from 1 to 21 days post-treatment. *Trichoderma asperelloides* (SuRDC-1442) and *T. canadense* (SuRDC-1422) also provided high MPDC (77–100%) from 1 to 21 days post-treatment against *N. parvum* (Table 4). The rest of the isolates provided lower MPDC (23–88%) against *N. parvum*. With the exception of *T. koningii* (SuRDC-1446), all isolates provided MPDC higher than 50% when pruning wounds were challenged with 5000 spores/wound of *D. seriata* and *N. parvum* 21 days post-treatment.

#### 3.3.2. Commercial Products Detached Cane Assay

Results from the DCA greenhouse trial assessing selected commercial products against these same *D. seriata* and *N. parvum* isolates are shown in Table 5. The average T and RH values for this DCA were determined to be 18.5 °C and 36% for the duration of the experiment in the greenhouse. MPRs of *D. seriata* and *N. parvum* from positive controls inoculated with 5000 spores/wound were higher than 73%, and pruning wound susceptibility decreased over time for both pathogens. *Diplodia seriata* and *N. parvum* were not isolated from the non-treated and non-inoculated (natural infection) negative controls. Tebuconazole provided lower MPDC for *D. seriata* (50–58%) than *N. parvum* (73–100%) from 1 to 21 days post-treatment. Tetraconazole provided even lower MPDC against *D. seriata* (29–39%) and practically no control against *N. parvum* (5–21%) from 1 to 21 days post-treatment. MPDC for both chemical products decreased over time. The biocontrol product based on a mixture of *T. asperellum* and *T. gamsii* showed an increase in MPDC over time with the highest control reached at 21 days post-treatment for both *D. seriata* (100%) and *N. parvum* (73%) (Table 5). *Trichoderma* species re-isolated from the detached cane assay were confirmed to be the same inoculated species via comparison of the ITS sequences, which showed 100% homology.

## 4. Discussion

This study represents the first attempt to characterize *Trichoderma* spp. naturally occurring on grapevines in Canada. Twenty-nine *Trichoderma* isolates were obtained from grapevines in BC and identified by sequencing the ITS gene and partial *TEF1* gene. In total, five known species, including *T. asperelloides*, *T. atroviride*, *T. harzianum*, *T. koningii*, and *T. tomentosum,* and two novel species, *T. canadense* and *T. viticola*, were identified. With over 350 species described, it has been shown that identification of *Trichoderma* based on ITS alone is challenging, as sequences are nearly identical among many species within the genus [47]. Therefore, several loci have been used in *Trichoderma* phylogenetic studies, including actin (*ACT*), calmodulin (*CaM1*), the second largest subunit of RNA polymerase II (*rpb2*), and *TEF1* [48,49,50]. Among them, *TEF1* has been shown to provide the highest resolution [47,49]. The *TEF1* phylogenetic analysis employed in this study allowed us to support species-level identification and agrees with previous studies on the use of *TEF1* alone to successfully identify *Trichoderma* spp. [28,41,51].

*Trichoderma asperelloides*, first described in 2010 as a cryptic species within *T. asperellum*, is known to have a wide sympatric distribution and has been isolated mostly from agricultural soils in tropical countries [52]. To the best of our knowledge, this is the first report of *T. asperelloides* in Canada and its first report from grapevines worldwide. *Trichoderma atroviride*, *T. harzianum*, and *T. koningii* are reported in many countries around the world from a broad range of different hosts, including grapevines [53]. In Canada, *T. atroviride* has been reported from different hosts and sources in Alberta, BC, Ontario (ON), and Québec (QC) provinces [54]. Similarly, *T. harzianum* has been found in Canada from *Cannabis sativa* [55], *Carpinus caroliniana* [56], *Populus tremuloides* [57], *Solanum lycopersocum* [58], and *Ulmus americana* [59]. On the other hand, *T. koningii* has a much narrower host range in Canada and has only been reported from *C. caroliniana* [56] and from mushroom compost [60]. *Trichoderma tomentosum*, first discovered in 1991 along with 11 other *Trichoderma* spp. as a species aggregate of *T. hamatum*, has been reported from *S. lycopersicum* in BC, *Helianthus annus* in Manitoba, *Ulmus* sp. in ON, and *Pinus* sp. in QC [61]. Since then, the species was shown to have a broader distribution and was reported from Europe [49], Guatemala [62], New Zealand [41], and the USA [61]. To the best of our knowledge, this is the first report of *T. tomentosum* from grapevines worldwide. In addition, this study identified several *Trichoderma* isolates in two distinct phylogenetic clades as representing two novel species named *T. canadense* and *T. viticola*, which are closely related to *T. koningiopsis* and *T. viride*, respectively. With seven different species identified from only 29 isolates collected, this study showed a high diversity of *Trichoderma* spp. in grapevines in BC. These results support further studies expanding the collection of *Trichoderma* isolates from grapevines and other hosts in BC, which could result in the identification of more species, and thus increase the possibility of finding effective BCAs.

This study evaluated the in vitro antagonism of 26 *Trichoderma* isolates against two BD pathogens: *D. seriata* and *N. parvum*. The only *T. tomentosum* isolate identified in this study (SuRDC-1431) did not survive in the culture collection and therefore was not included in this assay. *Diplodia seriata* and *N. parvum* pathogens were selected as they are reported to be among the most commonly isolated GTD fungi in BC [38]. In addition, they represent a moderately and a highly virulent species, respectively [9]. Previous studies have shown isolates of *T. asperelloides, T. atroviride*, *T. harzianum,* and *T. koningii* to be highly effective at inhibiting the growth of BD fungi, including *D. seriata* and *N. parvum,* in vitro [27,28,31], which is in agreement with our findings using isolates from BC. Although in vitro assays are not a guarantee of BCA activity in planta, assays such as the DCAA served as the first level of screening to identify which isolates exhibited the highest level of antagonism before investing in more advanced in planta screening, either under controlled greenhouse conditions or under natural field conditions. Results from our DCAA allowed us to select seven different isolates, belonging to six different species, to test them in planta alongside commercial products using a DCA under controlled greenhouse conditions. The DCA was first developed by Ayres et al. (2011) [46] to test the colonization of detached canes by *E. lata* over the course of three months, as well as to test the efficacy of grapevine pruning wound protection products against *E. lata*. This method eliminates the need for the potted vines used in other studies and allows for the inexpensive, high-throughput screening of pruning wound products in planta, which can yield rapid results [28], especially when compared against field trials, which most often span multiple years and may still yield inconclusive results [21,32,63]. Results from our DCA showed *Trichoderma* isolates SuRDC-1422 (*T. canadense*), SuRDC-1440 (*T. atroviride*), and SuRDC-1442 (*T. asperelloides*) to provide very high pruning wound protection when detached Chardonnay canes were treated immediately after pruning and then challenged with *D. seriata* or *N. parvum* one, seven, and twenty-one days later. These results were similar to a recent study screening *Trichoderma* isolates from different hosts in Italy against *D. seriata* and *N. parvum* using a DCA [28], which demonstrates the robust and repeatable nature of this evaluation system. The present study also evaluated for the first time the potential biocontrol activity of *T. koningii* and *T. asperelloides* against these GTD fungi.

*Trichoderma atroviride* is one of the most screened species for use against GTD fungi [26,28,31,32,64]. For instance, Pintos et al. [64] showed that treating pruning wounds with a *T. atroviride*-based commercial product resulted in reduction of *Botryosphaeriaceae* spp. recovery and necrosis lengths by 65.7% to 91.9%; however, the pathogen was still re-isolated from 38% of the *Trichoderma*-treated potted vines. Recently, Úrbez-Torres et al. [28] showed a *T. atroviride* isolate from tree fruit in Italy to reduce infection by *D. seriata* and *N. parvum* by 100% with no presence of the pathogen 21 d after treatment. In this study, *T. atroviride* isolate SuRDC-1440 was able to achieve a 93–100% reduction in pathogen recovery when challenged 24 h post-treatment and resulted in 100% MPDC of both *D. seriata* and *N. parvum* 7 and 21 days post-treatment. To date, only one study has reported the use of *T. asperelloides* against GTD fungi, showing high biocontrol activity against *Lasiodiplodia theobromae* [27]. Our study supports this finding of high biocontrol activity for *T. asperelloides* isolate SuRDC-1442 from BC, which resulted in 90–100% MPDC of *D. seriata* and *N. parvum* seven and twenty-one days post-treatment. Furthermore, this study shows for the first time high biocontrol activity from the novel species *T. canadense*, which can be compared to the activities of *T. atroviride* and *T. asperelloides*. This study also showed tebuconazole and *Trichoderma*-based commercial products to provide high pruning wound protection for *N. parvum* and both *D. seriata* and *N. parvum*, respectively. The efficacy of tebuconazole on the pruning wounds decreased over time while the efficacy of the commercial *Trichoderma*-based product increased significantly. Based on the results obtained in our DCAs, we can conclude that the *T. asperelloides*, *T. atroviride* and *T. canadense* isolates from BC provided similar or better pruning wound protection than the commercial products evaluated.

In previous studies, the inoculum dose used to evaluate the efficacy of either chemical fungicides or BCAs to protect pruning wounds from GTDs has varied greatly (10–100,000 conidia or ascospores per wound) [28,30,46]. In order to ensure robust recovery of the pathogens from the positive controls, wounds were challenged with 5000 conidia of either pathogen, which far exceeds the natural pressure that might be present in the field [13]. It was for this reason as well that we chose a Chardonnay cultivar, as it is known to be more susceptible to infection by GTD fungi, including *Botryosphaeriaceae* spp., than other cultivars [10,11]. With the combination of high inoculum pressure and a cultivar that is known to be susceptible, we were able to achieve high pathogen recovery from the positive controls in the DCA (73–100%). This is important to note because the value of MPDC reported is relative to the recovery of the pathogen, and further supports the high degree of biological control activity observed by the *Trichoderma* spp. in this study.

The three best-performing isolates in the DCA in this study, SuRDC-1422 (*T. canadense*), SuRDC-1440 (*T. atroviride*), and SuRDC-1442 (*T. asperelloides*), showed very high MPDC (63–100%) when *D. seriata* and *N. parvum* were inoculated only 24 h after treatment. These results were surprising, since *Trichoderma* is known to require time to colonize pruning wounds before becoming highly effective, as shown with the *Trichoderma*-based commercial product evaluated in this study. Therefore, studies evaluating *Trichoderma*-based products typically delay inoculation of pruning wounds with GTD fungi for up to seven days after treatment in order to give the product the best chance of success [30]. However, this is not ideal, as pruning wounds can be infected by GTD fungi immediately after pruning if spores are present in the environment [13]. Our results under greenhouse controlled conditions showed *Trichoderma* isolates from BC could potentially provide immediate pruning wound protection, similar to chemical fungicides but with longer-lasting and increasing effects. Similar results were obtained by Úrbez-Torres et al. (2020) [28] when evaluating *Trichoderma* isolates from tree fruits in Italy against the same BD fungi. It is possible that the optimal fungal growth conditions in the greenhouse while conducting the DCA favored the rapid expansion and colonization of pruning wounds by the different *Trichoderma* spp. However, this was not observed when evaluating the *Trichoderma*-based commercial product under the same experimental conditions. Therefore, we could hypothesize that significant differences may exist in the time it takes for *Trichoderma* to colonize the pruning wound depending on the source of the inoculum, in our case, raw spore-suspension versus formulated product. *Trichoderma* antifungal activity can be a result of direct competition for the same environmental niche or of the secreting of cell-wall-degrading enzymes and/or antagonistic secondary metabolites [17,18]. We cannot conclude which of these defense mechanisms are present in the *Trichoderma* spp. evaluated from BC, and further research could investigate the modes of action of antagonism by our isolates, as has been explored for other effective *Trichoderma* isolates [23,30].

In order to fully quantify the effectiveness of these *Trichoderma* isolates from BC, further screening is required in the field to evaluate their potential control under natural environmental conditions. Future work could also test for the tolerance of these effective isolates to fungicides, which may affect performance in the context of a field trial or commercial application management, where multiple fungicides may be used to control other grapevine diseases [27,51]. Similar to the DCA experiments, data are also needed regarding how long it takes for these *Trichoderma* spp. to protect the pruning wound and for how long they remain effective under natural field conditions, which will have important implications on developing a proper management strategy against BD fungi in the field.

## Figures and Tables

**Table 1:** Trichoderma isolates from grapevines of British Columbia used in this study.

		GenBank Accession No.	
Species	Isolate ^a^	ITS ^b^	TEF1 ^c^
*Trichoderma asperelloides*	SuRDC-1442/DAOMC 252428	MZ161800	MZ189381
*T. asperelloides*	SuRDC-1444/DAOMC 252430	MZ161801	MZ189382
*T. asperelloides*	SuRDC-1449/DAOMC 252434	MZ161802	MZ189383
*T. asperelloides*	SuRDC-1453/DAOMC 252436	MZ161803	MZ189384
*Trichoderma atroviride*	SuRDC-1424/DAOMC 252413	MZ161784	MZ189365
*T. atroviride*	SuRDC-1428/DAOMC 252417	MZ161785	MZ189366
*T. atroviride*	SuRDC-1432/DAOMC 252420	MZ161786	MZ189367
*T. atroviride*	SuRDC-1433/DAOMC 252421	MZ161787	MZ189368
*T. atroviride*	SuRDC-1436/DAOMC 252422	MZ161788	MZ189369
*T. atroviride*	SuRDC-1438/DAOMC 252424	MZ161789	MZ189370
*T. atroviride*	SuRDC-1440/DAOMC 252426	MZ161790	MZ189371
Trichoderma canadense *	SuRDC-1422/DAOMC 252411	MZ161796	MZ189377
*T. canadense*	SuRDC-1435	MZ161797	MZ189378
*T. canadense*	SuRDC-1450/DAOMC 2252435	MZ161798	MZ189379
*T. canadense*	SuRDC-1451	MZ161799	MZ189380
*Trichoderma* *harzianum*	SuRDC-1423/DAOMC 252412	MZ161805	MZ189386
*T. harzianum*	SuRDC-1425/DAOMC 252414	MZ161806	MZ189387
*T. harzianum*	SuRDC-1426/DAOMC 252415	MZ161807	MZ189388
*T. harzianum*	SuRDC-1437/DAOMC 252423	MZ161808	MZ189389
*T. harzianum*	SuRDC-1439/DAOMC 252425	MZ161809	MZ189390
*T. harzianum*	SuRDC-1441/DAOMC 252427	MZ161810	MZ189391
*T. harzianum*	SuRDC-1447/DAOMC 252432	MZ161811	MZ189392
*T. harzianum*	SuRDC-1448/DAOMC 252433	MZ161812	MZ189393
*Trichoderma* *koningii*	SuRDC-1443/DAOMC 252429	MZ161794	MZ189375
*T. koningii*	SuRDC-1446/DAOMC 252431	MZ161795	MZ189376
*Trichoderma tomentosum*	SuRDC-1431	MZ161804	MZ189385
*Trichoderma viticola*	SuRDC-1427/DAOMC 252416	MZ161791	MZ189372
*T. viticola*	SuRDC-1429/DAOMC 252418	MZ161792	MZ189373
T. viticola *	SuRDC-1430/DAOMC 252419	MZ161793	MZ189374

* Designated type specimen. ^a^ SuRDC: Summerland Research and Development Centre Fungal Collection, Summerland, BC, Canada. DAOMC: Department of Agriculture Ottawa Mycology Collection, Ottawa, ON, Canada. ^b^ Internal Transcribed Spacer (ITS1-5.8S-ITS2). ^c^ Translation elongation factor one-alpha.

**Table 2:** Commercial treatments, active ingredients and manufacturers of products evaluated for control of *Diplodia seriata* and *Neofusicoccum parvum* in detached cane assays.

Trade Name	Active Ingredients	Application Rate	Manufacturer
GreenSeal™ Ultra	Tebuconazole (10 g/L) in a paint form	n/a	Omnia Specialties Ltd. (Auckland, New Zealand)
Mettle^®^ 125 ME	Tetraconazole (125 g/L)	1.6 mL/L	Isagro Inc. (Durham NC, USA)
BIO-TAM^®^ 2.0	*T. asperellum* (2%) *+ T. gamsii* (2%)	96 g/L	Isagro Inc. (Durham NC, USA)

**Table 3:** Efficacy of *Trichoderma* isolates from BC applied immediately after pruning, followed by inoculation of 5000 conidia of *D. seriata* 1 day, 7 days, and 21 days post-treatment in a detached cane assay experiment.

Treatment	1 d.p.t.		7 d.p.t.		21 d.p.t.	
	MPR	MPDC	MPR	MPDC	MPR	MPDC
−Control	-	-	-	-	0	-
+Control	100 a	-	87 a	-	100 a	-
SuRDC-1422 (*T. canadense*)	33 bc	67	13 b	85	7 b	93
SuRDC-1427 (*T. viticola*)	77 a	23	20 cb	77	43 c	57
SuRDC-1437 (*T. harzianum*)	40 c	60	10 b	89	3 b	97
SuRDC-1439 (*T. harzianum*)	90 a	10	43 cd	50	13 b	87
SuRDC-1440 (*T. atroviride*)	7 b	93	0 b	100	0 b	100
SuRDC-1442 (*T. asperelloides*)	30 bc	70	3 b	96	0 b	100
SuRDC-1446 (*T. koningii*)	77 a	23	60 ad	31	53 c	47

d.p.t., days post-treatment. Values followed by the same letter(s) in each column were not statistically different using the Tukey–Kramer multiple comparison test (*p* = 0.05). MPR, *D. seriata* mean percent recovery. MPDC, mean percent disease control.

**Table 4:** Efficacy of *Trichoderma* isolates from BC applied immediately after pruning, followed by inoculation of 5000 conidia of *N. parvum* 1 day, 7 days, and 21 days post-treatment in a detached cane assay experiment.

Treatment	1 d.p.t.		7 d.p.t.		21 d.p.t.	
	MPR	MPDC	MPR	MPDC	MPR	MPDC
−Control	-	-	-	-	0	-
+Control	100 a	-	83 a	-	80 a	-
SuRDC-1422 (*T. canadense*)	23 bc	77	0 b	100	0 b	100
SuRDC-1427 (*T. viticola*)	37 bd	63	27 bc	68	13 bcd	83
SuRDC-1437 (*T. harzianum*)	60 de	40	57 ad	32	10 bc	88
SuRDC-1439 (*T. harzianum*)	77 ae	23	47 cd	44	37 d	54
SuRDC-1440 (*T. atroviride*)	0 c	100	0 b	100	0 b	100
SuRDC-1442 (*T. asperelloides*)	20 bc	80	0 b	100	3 bc	96
SuRDC-1446 (*T. koningii*)	3 bd	57	30 cd	64	27 cd	67

d.p.t., days post-treatment. Values followed by the same letter(s) in each column were not statistically different using the Tukey–Kramer multiple comparison test (*p* = 0.05). MPR, *N. parvum* mean percent recovery. MPDC, mean percent disease control.

**Table 5:** Efficacy of commercial products applied immediately after pruning, followed by inoculation of 5000 conidia of either *D. seriata* or *N. parvum* 1 day, 7 days, and 21 days post-treatment in a detached cane assay experiment.

	*Diplodia seriata*						*Neofusicoccum parvum*					
Treatment	1 d.p.t.		7 d.p.t.		21 d.p.t.		1 d.p.t.		7 d.p.t.		21 d.p.t.	
	MPR	MPDC	MPR	MPDC	MPR	MPDC	MPR	MPDC	MPR	MPDC	MPR	MPDC
−Control	-	-	-	-	0	-	-	-	-	-	0	-
+Control	87 a	-	83 a	-	80 a	-	97 a	-	87 a	-	73 a	-
Tebuconazole	37 c	58	47 b	44	40 b	50	0 c	100	7 b	92	20 b	73
Tetraconazole	53 bc	39	63 ab	24	57 ab	29	77 ab	21	83 a	4	70 a	5
*Trichoderma* spp.	70 ab	19	13 c	84	0 c	100	63 b	34	37 c	58	20 b	73

d.p.t., days post-treatment. Values followed by the same letter(s) in each column were not statistically different using the Tukey–Kramer multiple comparison test (*p* = 0.05). MPR, *D. seriata*/*N. parvum* mean percent recovery. MPDC, mean percent disease control.

## Data Availability

*Trichoderma* DNA sequences obtained in this study are available in GenBank (https://www.ncbi.nlm.nih.gov). *Trichoderma* isolates from British Columbia described in this study are available at the Summerland Research and Development Centre Fungal Collection (Summerland BC, Canada) and at the Canadian National Mycological Herbarium (DAOM, Ottawa, ON, Canada).
